# Bone mineral density and microarchitecture among Chinese patients with rheumatoid arthritis: a cross-sectional study with HRpQCT

**DOI:** 10.1186/s13075-021-02503-0

**Published:** 2021-04-24

**Authors:** Shangyi Jin, Mengtao Li, Qian Wang, Xiaofeng Zeng, Weibo Xia, Wei Yu, Wenmin Guan, Evelyn Hsieh

**Affiliations:** 1 Department of Rheumatology and Clinical Immunology, Chinese Academy of Medical Sciences & Peking Union Medical College; National Clinical Research Center for Dermatologic and Immunologic Diseases (NCRC-DID), Ministry of Science & Technology; State Key Laboratory of Complex Severe and Rare Diseases, Peking Union Medical College Hospital (PUMCH); Key Laboratory of Rheumatology and Clinical Immunology, Ministry of Education, Beijing, 100730 China; 2 Department of Endocrinology, Key Laboratory of Endocrinology, National Commission of Health, State Key Laboratory of Complex Severe and Rare Diseases, Peking Union Medical College Hospital, Chinese Academy of Medical Sciences, Beijing, 100730 China; 3 Department of Radiology, Peking Union Medical College Hospital, Chinese Academy of Medical Sciences and Peking Union Medical College, Beijing, China; 4 Section of Rheumatology, Allergy and Immunology, Yale School of Medicine, 300 Cedar Street, TAC S-525, P.O. Box 208031, New Haven, CT 06520-8031 USA

**Keywords:** Rheumatoid arthritis, Bone mineral density, Microarchitecture, HRpQCT, Fragility fracture

## Abstract

**Background:**

Patients with rheumatoid arthritis (RA) are at increased risk of fractures. Although their decline in bone mineral density (BMD) is well-established, data regarding the alterations in bone microarchitecture are limited. In this study, we aimed to evaluate bone microarchitecture, geometry, and volumetric BMD among patients with RA in mainland China using high-resolution peripheral quantitative computed tomography (HRpQCT).

**Methods:**

In this cross-sectional study, patients with RA were recruited from the Peking Union Medical College Hospital site of the Chinese Registry of rhEumatoiD arthrITis (CREDIT). Each participant underwent HRpQCT scanning (Scanco XtremeCT II), thoracolumbar X-ray and dual-energy X-ray absorptiometry. The primary outcomes were HRpQCT-related measures at distal radius and tibia. Data regarding demographic features, RA-related characteristics, and history of fragility fractures were collected. Correlation between HRpQCT parameters and potentially related factors were analyzed using linear regression analysis. A group of age- and sex-matched healthy controls was included for comparison.

**Results:**

A total of 81 patients with RA [69 women, aged 57.9 ± 8.7 years, disease duration 5.7 (IQR 1.4–11.2) years] and 81 matched healthy controls were included. Compared with controls, patients with RA had significantly larger bone area and lower total and trabecular vBMD at both the distal radius and tibia. Lower cortical bone thickness was also shown at the distal tibia. Among patients with RA, advanced age, low BMI, female sex, disease duration, and activity were associated with decreased vBMD and impaired bone microstructure. Female reproductive factors including menopause, late menarche, breast feeding, and early childbirth also showed negative correlation with these parameters. Compared to patients with RA without fractures, patients with fragility fractures (*n* = 11) showed lower trabecular and cortical vBMD, thinner cortical bone, impaired trabecular microstructure, and a trend of declined bone strength. Current glucocorticoid intake was related to decreased vBMD, trabecular number, increased trabecular separation, and inhomogeneity.

**Conclusions:**

In this study, we observed alterations in bone mineral density, geometry, and microarchitecture among patients with RA compared to healthy individuals, which may impair bone strength and lead to increased risk of fractures. Both traditional risk factors for osteoporosis and RA-associated factors need to be considered in the assessment of the bone quality.

**Supplementary Information:**

The online version contains supplementary material available at 10.1186/s13075-021-02503-0.

## Background

Rheumatoid arthritis (RA) is a common autoimmune disease characterized by chronic symmetrical polyarthritis and various extra-articular manifestations [1]. In addition to periarticular osteopenia and bone erosion, patients with RA may also develop generalized bone loss and have increased risk for fractures [2, 3]. Indeed, the fracture risk assessment tool (FRAX), an internationally validated tool for fracture risk prediction, includes RA as an independent risk factor for 10-year probability of osteoporotic fractures [4]. Drivers of risk for fracture in RA are multifactorial [5], and fractures can lead to significant morbidity and even increased mortality [6, 7]. Since timely intervention could help prevent subsequent fractures, assessment of fracture risk is of vital importance in the long-term management of patients with RA [8].

In clinical practice, assessment of fracture risk is currently mainly based on patients’ demographic features, clinical characteristics, and areal bone mineral density (aBMD) as measured by dual X-ray absorptiometry (DXA). However, it has been shown that declines in BMD may only explain 70% of impairment in bone strength [9], and some fractures occur among patients with normal BMD or osteopenia [10]. Other determinants of the bone strength include bone microarchitecture, geometry, mineralization, and bone turnover [11]. A better understanding of the role of these determinants may help elucidate the mechanisms of systemic bone loss and fracture in RA and improve clinical evaluation of a fracture risk.

High-resolution peripheral quantitative computed tomography (HRpQCT) is a new and non-invasive radiographic technique which can simultaneously assess bone microarchitecture, volumetric BMD, bone geometry, and biomechanical properties [12]. To date, it has been primarily applied in research settings to examine bone health in the general population, post-menopausal women, and patients with secondary osteoporosis [13–16], showing its potential use in mechanistic research, fracture prediction, and treatment evaluation [17]. HRpQCT has also recently been applied to study rheumatoid arthritis, revealing the adverse impact of this disease on bone density, microstructure, and strength properties at both the hands and distal radius of patients [18–22]. However, alterations in the bone at sites distant from inflamed joints, which may better represent the generalized bone loss in RA, are still undefined. In addition, data is scarce regarding the correlation between HRpQCT parameters and fracture, the outcome of greatest clinical importance in studies of bone health.

To address this gap, we designed a cross-sectional study utilizing HRpQCT to explore bone alterations at both the distal radius and tibia among patients with RA at a large tertiary care hospital in Beijing, China, and we also aimed to identify potential demographic and clinical factors associated with these alterations.

## Methods

### Study design

In this cross-sectional study, patients with RA receiving routine care at the Peking Union Medical College Hospital (PUMCH) were recruited. For each participant, HRpQCT parameters were measured at both the distal radius and distal tibia and compared with an age and sex-matched group of healthy controls. Correlation between HRpQCT parameters with patients’ demographics, RA-related characteristics, fracture history, and other risk factors for osteoporosis were also analyzed.

### Study population

#### RA group

Established in Nov 2016, the Chinese Registry of rhEumatoiD arthrITis (CREDIT) is the first nationwide, multicenter prospective registry of rheumatoid arthritis patients in China [23, 24]. Consecutive patients visiting participating centers were invited to enroll in the registry if they fulfilled the 2010 American College of Rheumatology classification criteria for RA [25]. In the present study, from January to June 2018, all patients with RA in the PUMCH CREDIT database were considered eligible for inclusion and were invited to participate when they presented for routine clinical care. In addition, we purposefully sampled patients with a history of fracture by inviting all patients in the database who reported having an osteoporotic fracture after onset of RA. Exclusion criteria included pregnancy and history of fracture prior to RA diagnosis. Ethics approvals for all surveys and measurements were obtained from the Institutional Review Board of PUMCH prior to study initiation. Written informed consent was obtained from all patients at the time of enrollment in CREDIT and additionally at the time of HRpQCT measurement.

#### Healthy control group

A group of age- and sex-matched healthy controls were randomly selected from a cohort of healthy individuals recruited by the endocrinology department of PUMCH from June 2015 to February 2017, as described in a previous study [26]. The cohort was comprised of PUMCH students, staff, and people presenting to the PUMCH for physical examination. Exclusion criteria included history of hyperparathyroidism, hyperthyroidism, hypothyroidism, rheumatologic disease, chronic liver or kidney disease, osteogenesis imperfecta, ovariectomy, use of hormone replacement therapy, prior treatment with glucocorticoids, and anti-osteoporotic agents. All participants completed a brief questionnaire regarding personal characteristics and obtained anthropometric data and HRpQCT imaging.

### Sociodemographic and clinical characteristics

All study participants in the RA group completed an electronic questionnaire focused on the following information: demographic data, RA disease history [disease duration, disease activity measured by disease activity score 28 (DAS28), Simplified Disease Activity Index (SDAI) and Clinical Disease Activity Index (CDAI), positivity for rheumatoid factor (RF) and anti-citrullinated protein antibodies (anti-CCP), joint function assessment by Health Assessment Questionnaire Disability Index (HAQ-DI), past and present treatment, as well as the presence of major comorbidities], risk factors for osteoporosis (smoking, alcohol use, parental history of fractures, personal history of fractures, secondary osteoporosis, use of glucocorticoids, history of falls in the past year and for women, reproductive history), and bone health history (previous BMD evaluation, physical activity, anti-osteoporotic therapies, calcium, and vitamin D dietary intake and supplements). Disease-related data were collected by patients’ treating rheumatologists, who had been trained for clinical assessment and data collection according to a predefined protocol, and completion of the study questionnaire was guided and supervised by the same investigator for all participants.

### Ascertainment of fractures

(1) Self-reported data were collected regarding fragility fractures (also called osteoporotic fractures, OPFs), which were defined as fractures resulting from minor injuries (i.e., a fall from standing height or less) or occurring during daily activities. Fractures due to high-impact injuries such as motor vehicle accidents and athletic activities were excluded. (2) Lateral thoracolumbar X-ray was performed for each patient, and the presence of vertebral fractures was determined using the semi-quantitative technique described by Genant et al. [27]. All X-rays were reviewed in a blinded fashion by the same reader, with over 40 years of experience in this methodology. In this study, vertebral deformities of grades 2 and 3 (moderate and severe deformities) were defined as radiographic vertebral fractures [16].

### DXA measures

Areal BMD (aBMD) of the lumbar spine (L1–L4) and left hip (total hip and femoral neck) was obtained for each participant by a trained technician using the same DXA equipment (GE Lunar Prodigy Advanced scanner, GE Healthcare, Madison, WI) and software (enCORE version 10.50.086). Results were expressed in g/cm^2^, and T scores were calculated with reference to normal Chinese young adults. Osteoporosis was defined according to the World Health Organization classification [28].

### HRpQCT measures

HRpQCT were performed at the non-dominant distal radius and tibia of all participants using the second generation HRpQCT scanner (XtremeCT II, Scanco Medical AG, Bruttisellen, Switzerland), except in cases of previous fracture or surgery when the contralateral side was measured. Subjects were positioned with their wrists and ankles immobilized in a carbon-fiber cast from the manufacturer and asked to refrain from talking for at least 2 min. The reference line was set manually at the end plates of distal radius and tibia, and measurements were carried out 9 mm and 22 mm proximal to the reference lines, respectively, and spanned proximally 9.02 mm in length. The standard mode (68kVp, 1462 μA, 100 ms, with an isotropic resolution of 61 μm) was used, and for each site, 168 slices were acquired and analyzed. All scans were completed by a trained technologist according to the manufacturer’s protocol.

### Postprocessing of HRpQCT images

For each scan, standard semi-automated analysis software was used to determine the following parameters: (1) *Bone geometry:* total bone cross-sectional area (Tt.Ar), cortical perimeter (Ct.Pm), cortical area (Ct.Ar), and trabecular area (Tb.Ar). (2) *Volumetric bone mineral densities:* total volumetric bone mineral density (Tt.vBMD), trabecular volumetric bone density (Tb.vBMD), and cortical volumetric density (Ct.vBMD). (3) *Microarchitecture:* trabecular bone volume fraction (BV/TV), trabecular number (Tb.N), trabecular thickness (Tb.Th), trabecular separation (Tb.Sp), inhomogeneity of the network (Tb.1/N.SD), cortical thickness (Ct.Th), and cortical porosity (Ct.Po).

Micro-finite element analyses (μFEA) were conducted with the Scanco Finite Element software (vision 1.13, Scanco Medical). The binary image was turned into a mesh of isotropic brick elements, and a uniaxial compression test with a 1000 N load was performed with 1% apparent strain. All elements were assigned a homogenous elastic modulus of 10 GPa and a Poisson’s ratio of 0.3. Failure load was assumed to occur when 2% of the elements exceed a local effective strain of 0.7% [29].

### Statistical analysis

Descriptive statistics were used to present the demographics, clinical characteristics, and HRpQCT parameters of patients with RA and matched healthy controls. Normally distributed continuous variables were presented as mean and standard deviation, and non-normally distributed variables were presented as median and interquartile range. Categorical variables were presented as proportions. Stratified analyses were used to evaluate the impact of selected key characteristics on HRpQCT parameters. Continuous variables were compared using the Student’s *t* test and Mann-Whitney *U* tests, and chi-squared tests were used for categorical variables. The correlation between continuous variables was analyzed using Pearson or Spearman correlation coefficients according to data distribution. Univariable and multivariable linear regression analyses were applied in the RA group to identify potential factors associated with HRpQCT parameters. Variables of clinical importance in previous studies including age, sex, BMI, RA duration and disease activity, and factors with *p* value<0.1 in the univariable analysis were included in multivariable analyses.

Statistical significance was defined as *p* < 0.05. All statistical analyses were performed using SPSS software (version 23.0, IBM SPSS Inc., Armonk, NY, USA).

## Results

### Characteristics of participants

A total of 81 patients with RA and 81 age- and sex-matched healthy controls [mean age 57.9 vs 58.3 years (*p* = 0.791), and 69 women in both groups] were included (a flowchart of study inclusion is shown in Supplemental Figure 1). No significant differences were found between the RA and healthy control groups in terms of height, weight, BMI, and for women, in menopausal status. Demographic data and clinical characteristics of all participants are shown in Table 1.

**Table 1:** Characteristics of patients with RA and healthy controls

Characteristic	RA *n* = 81	Control group *n* = 81
Age, years	57.9 ± 8.7	58.3 ± 8.8
Female, *n* (%)	69 (85.2)	69 (85.2)
Post-menopausal, *n* (%)	52 (75.4)	51 (73.9)
Height, cm	160.5 ± 7.0	160.8 ± 7.1
Weight, kg	62.5 ± 10.9	60.1 ± 10.7
BMI, kg/m^2^	24.2 ± 3.5	23.2 ± 3.4
Ever smoker, *n* (%)	17 (21.0)	–
Alcohol use, *n* (%)	18 (22.2)	–
Parental hip fracture, *n* (%)	9/77 (11.7)	–
Fall in the past year, *n* (%)	18/78 (23.1)	–
Duration of RA, years	5.7 (1.4–11.2)	–
RF or ACPA+, *n* (%)	74 (91.4)	–
ACPA+, *n* (%)^a^	53 (84.1)	–
DAS28-ESR	3.4 ± 1.5	–
DAS28-CRP	2.9 ± 1.4	–
SDAI	8.2 (3.2–16.2)	–
CDAI	7.0 (3.0–15.8)	–
HAQ-DI	0.05 (0.00–0.28)	–
GC ever, *n* (%)	55 (67.9)	–
Current dose, mg/day	5.6 (5.0–10.0)	–
Maximum dose, mg/day	10.0 (10.0–21.2)	–
Duration, m	17.0 (6.5–58.0)	–
csDMARDs, *n* (%)	74 (91.4)	–
MTX ever, *n* (%)	57 (70.4)	–
LEF ever, *n* (%)	31 (38.3)	–
Sulfasalazine ever, *n* (%)	4 (4.9)	–
HCQ ever, *n* (%)	20 (24.7)	–
TWH ever, n(%)	29 (35.8)	–
bDMARDs ever, *n* (%)	14 (17.3)	–
Bisphosphonates, *n* (%)	7 (8.6)	0(0)

Data regarding demographic features, RA-related characteristics and past and present treatment of patients with RA and age- and sex-matched healthy controls are presented in this table

*RA* rheumatoid arthritis, *RF* rheumatoid factor, *ACPA* anti-citrullinated protein antibodies, *ESR* erythrocyte sedimentation rate, *CRP* C-reactive protein, *DAS28* disease activity score 28, *CDAI* Clinical Disease Activity Index, *SDAI* Simplified Disease Activity Index, *HAQ-DI* Health Assessment Questionnaire Disability Index, *GC* glucocorticoid, *MTX* methotrexate, *LEF* leflunomide, *csDMARD* conventional synthetic DMARD, *HCQ* hydroxychloroquine, *bDMARD* biological DMARD, *OP* osteoporosis, *TWH* tripterygium wilfordii Hook

^a^Data on ACPA were missing from 18 patients

### Geometry, vBMD, and bone microarchitecture in RA

As is shown in Table 2, compared with healthy controls, patients with RA had significantly larger total and trabecular bone area, increased cortical bone perimeter, and lower total and cortical vBMD at the distal radius. They also had lower total vBMD and thinner cortical bone at the distal tibia.

**Table 2:** HRpQCT parameters among patients with and without RA

**Distal radius**	**RA group (*n*** **= 81)**	**Control group (*n*** **= 81)**	**Difference**	***P***
**Geometry**				
Tt.Ar, mm^2^	280.8 ± 66.9	253.1 ± 51.5	10.9%	**0.004**
Ct.Ar, mm^2^	57.5 ± 13.8	56.2 ± 11.1	2.3%	0.506
Tb.Ar, mm^2^	227.2 ± 64.6	200.425 ± 48.5	13.4%	**0.003**
Ct.Pm, mm^2^	71.0 ± 9.8	66.3 ± 7.5	7.1%	**0.001**
**Volumetric BMD, mgHA/ccm**				
Tt.vBMD	258.5 ± 83.2	284.3 ± 70.2	−9.1%	**0.034**
Ct.vBMD	882.9 ± 111.6	911.7 ± 58.7	−3.2%	**0.042**
Tb.vBMD	94.5 ± 42.7	106.6 ± 42.4	−11.4%	0.071
**Microarchitecture**				
BV/TV	0.144 ± 0.056	0.160 ± 0.057	−10.0%	0.073
Tb.Th, mm	0.219 ± 0.019	0.219 ± 0.016	0.0%	0.864
Tb.N, 1/mm	1.087 ± 0.326	1.148 ± 0.295	−5.3%	0.215
Tb.Sp, mm	1.042 ± 0.538	0.931 ± 0.393	11.9%	0.136
Tb.1/N.SD	0.360 (0.284–0.508)	0.318 (0.268–0.508)	13.2%	0.155
Ct.Th, mm	0.978 ± 0.256	1.006 ± 0.188	−2.8%	0.431
Ct.Po, %	0.60 (0.30–1.00)	0.50 (0.30–0.85)	20.0%	0.279
**Distal tibia**	**RA group (*n*** **= 80)**	**Control group (*n*** **= 81)**	**Difference**	***P***
**Geometry**				
Tt.Ar, mm^2^	712.4 ± 128.5	679.3 ± 110.4	4.9%	0.081
Ct.Ar, mm^2^	101.9 ± 28.1	107.4 ± 21.8	−5.1%	0.164
Tb.Ar, mm^2^	615.6 ± 124.3	577.2 ± 110.0	6.7%	**0.039**
Ct.Pm, mm^2^	103.9 ± 9.5	101.3 ± 8.5	2.6%	0.071
**Volumetric BMD, mgHA/ccm**				
Tt.vBMD	218.1 ± 63.5	237.0 ± 51.8	−8.0%	**0.040**
Ct.vBMD	859.8 ± 89.8	869.5 ± 77.4	−1.1%	0.465
Tb.vBMD	109.3 ± 36.8	117.1 ± 36.5	−6.7%	0.179
**Microarchitecture**				
BV/TV	0.179 ± 0.047	0.189 ± 0.047	−5.3%	0.196
Tb.Th, mm	0.241 ± 0.019	0.240 ± 0.019	0.4%	0.627
Tb.N, 1/mm	1.116 ± 0.234	1.134 ± 0.207	−1.6%	0.608
Tb.Sp, mm	0.939 ± 0.239	0.902 ± 0.194	4.1%	0.281
Tb.1/N.SD	0.360 (0.284–0.508)	0.318 (0.268–0.508)	13.2%	0.398
Ct.Th, mm	1.165 ± 0.307	1.255 ± 0.264	−7.2%	**0.048**
Ct.Po, %	2.45 (1.40–3.95)	3.00 (1.45–3.90)	−16.7%	0.320

HRpQCT measures at the radius and tibia among patients and healthy controls are presented, organized by geometric, volumetric, and microarchitectural parameters. Comparisons of each measure (mean ± SD) between patients with RA and health controls were calculated using the Student’s *t* test

*Tt.Ar* total bone area, *Ct.Ar* cortical bone area, *Tb.Ar* trabecular bone area, *Ct.Pm* cortical perimeter, *Tt.vBMD* total volumetric BMD, *Ct.vBMD* cortical vBMD, *Tb.vBMD* trabecular vBMD, *BV/TV* trabecular bone volume fraction, *Tb.Th* trabecular thickness, *Tb.N* trabecular number, *Tb.Sp* trabecular separation, *Tb.1/N.SD* inhomogeneity of network, *Ct.Th* cortical thickness, *Ct.Po* intra-cortical porosity

### Impact of sex and reproductive factors on HRpQCT parameters

In both healthy control and RA groups, men had larger bone size than women (Supplemental Table 1). Compared with healthy women, healthy men had higher trabecular vBMD, trabecular bone volume fraction and number, lower trabecular separation, thicker trabecular, and cortical bone as well as higher cortical porosity. Sex differences in the RA group shared the same tendency as healthy controls, but without statistical significance. For women with RA, menopause adversely influenced almost all parameters (Supplemental Table 2). Late age at menarche was related to lower vBMD and impaired trabecular microstructure. Earlier age at first childbirth and more children are related to decreased vBMD, thinner trabecular, and cortical bone as well as inhomogeneity of the tibial trabeculae.

### Impact of history of fragility fractures

**Fig. 1:**
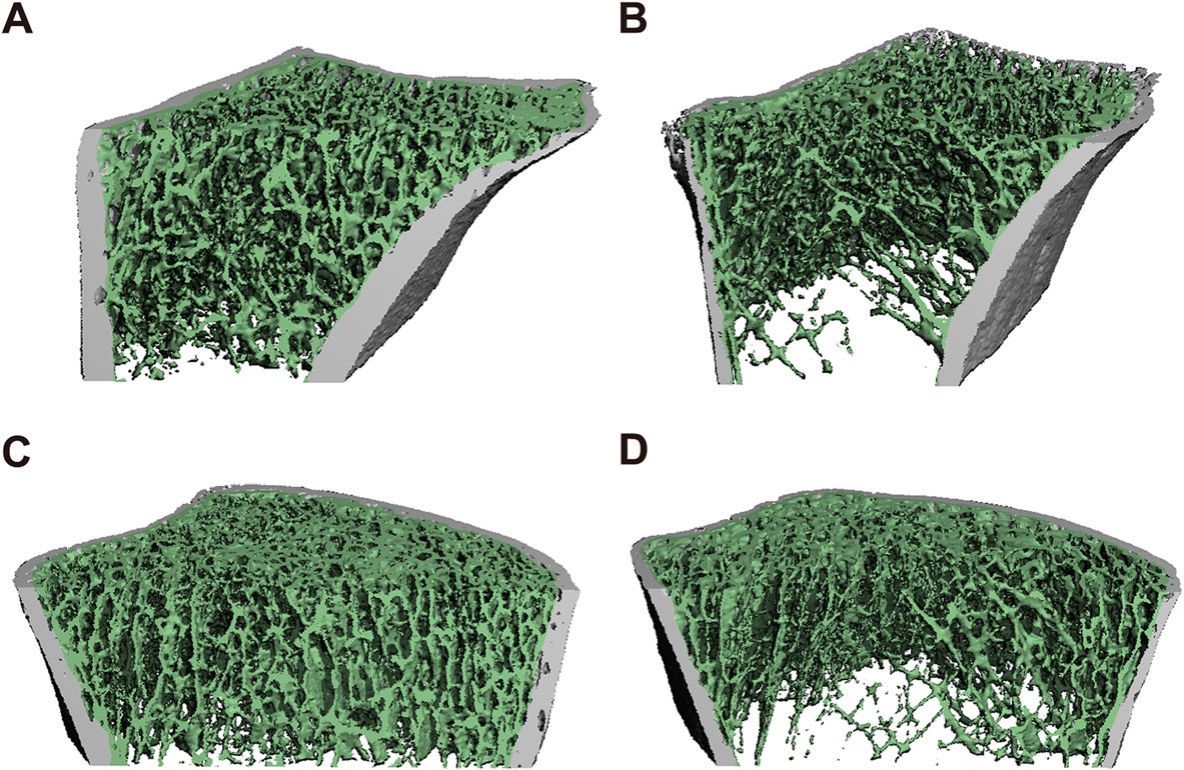
Three-dimensional reconstruction images of a pair of age- and sex-matched patients with RA, with (right column) and without (left column) history of fragility fractures; distal radius (top) and distal tibia (bottom). Compared with patients without fractures, patients with fragility fractures have generalized alterations in both trabecular and cortical bone at the distal radius and tibia

Eleven patients (62.5 ± 6.7 years, 10 females) in the RA group were identified as having moderate to severe fragility fractures after onset of RA based upon X-rays and/or clinical history. A total of 26 fractures were identified, including 24 major osteoporotic fractures [at vertebrae (*n* = 21), hip (*n* = 1), forearm (*n* = 1), and shoulder (*n* = 1)] and 2 at other sites (1 at ribs and 1 at patella). Patients with and without fragility fractures had no significant differences in age or sex. HRpQCT of the fracture group showed lower vBMD at both the distal radius and tibia. Impaired bone microstructure including thinner cortical bone, decreased trabecular number, larger trabecular bone separation, and increased inhomogeneity was also presented (Table 3 and Fig. 1). μFEA was performed for the 11 patients with fractures and 11 age- and sex-matched patients without fractures in the RA group. Patients with fractures showed a trend of lower stiffness and failure load at both bone sites (Table 3).

**Table 3:** HRpQCT parameters among patients with RA, by fracture status

	Distal radius			Distal tibia		
	Fracture *n* = 11	No fracture *n* = 70	*P*	Fracture *N* = 11	No fracture *N* = 70	*P*
**Geometry**						
Tt.Ar, mm^2^	312.2 ± 89.0	275.9 ± 62.1	0.095	755.3 ± 135.4	705.6 ± 127.1	0.236
Ct.Ar, mm^2^	54.4 ± 15.3	58.0 ± 13.7	0.422	89.8 ± 24.3	103.8 ± 28.3	0.125
Tb.Ar, mm^2^	262.8 ± 91.1	221.6 ± 58.3	**0.049**	671.1 ± 130.6	606.8 ± 122.0	0.112
Ct.Pm, mm	77.9 ± 13.4	70.0 ± 8.8	**0.012**	107.3 ± 9.6	103.3 ± 9.4	0.204
**Volumetric BMD, mgHA/ccm**						
Tt.vBMD	195.5 ± 81.1	268.4 ± 79.6	**0.006**	163.0 ± 45.9	226.9 ± 61.7	**0.002**
Ct.vBMD	765.3 ± 180.7	901.4 ± 84.7	**0.032**	801.8 ± 76.7	869.0 ± 88.8	**0.020**
Tb.vBMD	68.6 ± 41.5	98.5 ± 41.8	**0.030**	78.8 ± 28.4	114.1 ± 35.8	**0.003**
**Microarchitecture**						
BV/TV	0.117 ± 0.049	0.149 ± 0.056	0.076	0.143 ± 0.034	0.185 ± 0.047	**0.006**
Tb.Th, mm	0.220 ± 0.016	0.219 ± 0.020	0.795	0.243 ± 0.019	0.241 ± 0.019	0.698
Tb.N, 1/mm	0.838 ± 0.336	1.126 ± 0.309	**0.006**	0.903 ± 0.255	1.150 ± 0.214	**0.001**
Tb.Sp, mm	1.089(0.892–1.641)	0.875(0.715–1.074)	**0.014**	1.210(0.841–1.503)	0.849(0.767–1.037)	**0.005**
Tb.1/N.SD	0.522(0.429–1.179)	0.349(0.280–0.478)	**0.004**	0.539(0.324–0.975)	0.353(0.287–0.466)	**0.007**
Ct.Th, mm	0.854 ± 0.370	0.997 ± 0.231	0.084	0.988 ± 0.277	1.194 ± 0.304	**0.039**
Ct.Po,%	0.60(0.40–1.50)	0.30(0.60–0.90)	0.427	2.50(1.60–4.00)	2.30(1.25–3.95)	0.670
μFEA*						
Stiffness, kN/mm	41.8 ± 19.0	46.6 ± 16.2	0.540	109.8 ± 25.3	131.2 ± 40.9	0.155
Failure Load, N	2201 ± 982	2506 ± 937	0.476	6118 ± 1378	7233 ± 2277	0.180

HRpQCT parameters at the distal radius and tibia were compared between patients with and without fractures in the RA group. Significant differences were shown in parameters regarding bone geometry, BMD, and microarchitecture

*Performed in 11 patients with fracture and 11 age-and sex-matched patients without fracture

### Influence of treatment with glucocorticoids

History of any systemic treatment with GCs showed no correlation with HRpQCT measures, and we further analyzed whether current treatment, dosage, or duration of GCs were related. Current treatment with GCs was correlated with decreased cortical vBMD and higher porosity at both bone sites (Supplemental Table 3). They also had decreased trabecular number, increased trabecular separation, and inhomogeneity at distal tibia. No correlations were observed with regards to dose or duration of GCs.

### Correlation between DXA and HRpQCT parameters

As is shown in Table 4, aBMD measured by DXA at both the lumbar spine and total hip were positively correlated with vBMD, trabecular bone volume fraction, trabecular number, and cortical thickness and was negatively correlated with trabecular separation and inhomogeneity. Trabecular thickness and cortical porosity were not correlated with aBMD at any sites.

**Table 4:** Correlation analyses between HRpQCT parameters and aBMD, *n* = 81

	**vBMD**			**BV/TV**	**Tb.Th**	**Tb.N**	**Tb.Sp**	**Inhomogeneity**	**Ct.Th**	**Ct.Po**
	Tt	Tb	Ct							
**Distal radius**										
aBMD at Lumbar spine	0.712*	0.614*	0.552*	0.556*	0.144	0.644*	−0.521*	−0.483*	0.636*	−0.029
aBMD at Femoral neck	0.652*	0.593*	0.497*	0.558*	0.209	0.600*	−0.486*	− 0.467*	0.630*	0.052
aBME at Total hip	0.686*	0.635*	0.541*	0.600*	0.187	0.662*	−0.536*	− 0.527*	0.633*	0.001
**Distal tibia**										
aBMD at Lumbar spine	0.733*	0.637*	0.572*	0.589*	0.020	0.651*	−0.667*	−0.610*	0.607*	−0.101
aBMD at Femoral neck	0.708*	0.606*	0.506*	0.582*	0.164	0.552*	−0.524*	− 0.470*	0.666*	− 0.025
aBMD at Total hip	0.803*	0.689*	0.556*	0.665*	0.216	0.607*	−0.595*	−0.532*	0.740*	−0.027

Correlation exists between the majority of HRpQCT parameters and aBMD of axial skeleton measured with DXA, with the exception of Ct.Po and Tb.Th. Results are presented with Pearson or Spearman correlation coefficients

*p<0.05

### Factors associated with HRpQCT parameters among patients with RA

According to the multivariable analyses of bone geometry, female sex was associated with smaller trabecular and cortical bone area at both the distal radius and tibia (Tables 5 and 6). Trabecular area at distal radius showed a positive correlation with disease duration (*p* = 0.012) and functional disability (*p* = 0.044), while ever use of HCQ had negative association with it (*p* = 0.028). At distal tibia, trabecular area was correlated with advanced age (*p* = 0.004). The cortical area was negatively correlated with advanced age and low BMI at both sites.

**Table 5:** Multivariable linear regression analysis of factors associated with HRpQCT parameters at the distal radius among patients with RA, *n* = 81

**Distal radius**	**Tb.Ar (mm**^**2**^**)**		**Ct.Ar (mm**^**2**^**)**		**Tt.vBMD**		**Tb.vBMD**		**Ct.vBMD**		**Ct.Th (mm)**	
	*β*	*P*	*β*	*P*	*β*	*P*	*β*	*P*	*β*	*P*	*β*	*P*
Age, years	0.904	0.211	−0.556	**0.000**	−4.145	**0.000**	−1.397	**0.010**	−4.635	**0.000**	−0.012	**0.000**
Female	−80.386	**0.000**	−20.183	**0.000**	−19.657	0.341	−33.415	**0.005**	30.57	0.262	−0.156	**0.021**
BMI	−0.633	0.708	1.114	**0.002**	3.846	0.062	3.121	**0.009**	2.88	0.288	0.018	**0.009**
Grip strength	1.529	0.112	0.157	0.426	–	–	–	–	–	–	–	–
Duration, years	1.882	**0.012**	0.059	0.694	−2.526	**0.010**	−1.394	**0.010**	−4.545	**0.001**	−0.002	0.424
DAS28	4.906	0.275	1.189	0.200	−5.802	0.285	−1.669	0.549	−14.252	**0.049**	−0.002	0.892
HAQ	24.828	**0.044**	−4.068	0.108	−11.831	0.458	–	–	−31.548	0.136	−0.108	**0.037**
GC ever	–	–	–	–	–	–	–	–	− 14.244	0.479	–	–
HCQ	−29.553	**0.028**	4.546	0.099	42.453	**0.016**	16.638	0.099	–	–	0.091	0.106
**Distal radius**	**Tb.Th (μm)**		**Tb.N**		**Tb.Sp (mm)**		**Tb.1/N.SD**		**BV/TV**		**Ct.Po (%)**	
	*β*	*P*	*β*	*P*	*β*	*P*	*β*	*P*	*β*	*P*	*β*	*P*
Age, years	−0.144	0.584	−0.010	**0.008**	0.011	0.082	0.009	0.109	−0.002	**0.020**	0.005	0.540
Female	−15.973	**0.011**	−0.119	0.273	0.291	0.055	0.217	0.094	−0.042	**0.007**	−0.720	**0.001**
BMI	0.536	0.393	0.020	**0.038**	−0.029	0.058	− 0.029	**0.031**	0.004	**0.006**	0.048	**0.023**
Grip strength	–	–	0.010	0.052	–	–	–	–	–	–	–	–
Duration, years	−0.126	0.651	−0.009	**0.017**	0.015	**0.030**	0.010	0.098	−0.002	**0.017**	0.016	0.086
DAS28	1.501	0.312	−0.005	0.833	0.108	**0.004**	0.074	**0.023**	−0.002	0.671	−0.006	0.903
HCQ	–	–	–	–	–	–	–	–	0.026	0.053	–	–
bDMARD	–	–	−0.094	0.250	–	–	0.24	0.055	–	–	–	–
TWH	–	–	–	–	–	–	–	–	–	–	0.311	**0.042**

Multivariable linear regression analysis was performed to identify potential factors associated with HRpQCT parameters at the distal radius. Results for different parameters are presented in separate columns. *β β*-coefficient, *TWH* tripterygium wilfordii Hook

**Table 6:** Multivariable linear regression analysis of factors associated with HRpQCT parameters at the distal tibia among patients with RA, *n* = 81

**Distal tibia**	**Tb.Ar (mm**^**2**^**)**		**Ct.Ar (mm**^**2**^**)**		**Tt.vBMD**		**Tb.vBMD**		**Ct.vBMD**		**Ct.Th (mm)**	
	*β*	*P*	*β*	*P*	*β*	*P*	*β*	*P*	*β*	*P*	*β*	*P*
	4.586	**0.004**	−1.059	**0.000**	−3.044	**0.000**	−0.823	0.083	−6.256	**0.000**	−0.012	**0.002**
Female	−112.593	**0.009**	−32.411	**0.000**	−30.981	0.064	−22.203	**0.045**	10.934	0.637	−0.288	**0.001**
BMI	−0.377	0.916	2.358	**0.001**	5.277	**0.002**	3.204	**0.005**	4.349	0.060	0.027	**0.002**
Grip strength	3.594	0.080	0.723	0.072	–	–	–	–	–	–	–	–
Duration, years	−0.367	0.806	−0.138	0.648	−1.351	0.077	−0.792	0.110	−0.232	0.829	−0.004	0.360
DAS28	11.27	0.202	3.131	0.097	−1.002	0.803	−1.041	0.692	−0.680	0.911	0.020	0.375
HAQ	–	–	−7.507	0.142	–	–	–	–	−11.368	0.525	−0.070	0.289
	−17.152	0.543	–	–	28.834	**0.046**	–	–	9.501	0.625	0.106	0.140
**Distal tibia**	**Tb.Th (μm)**		**Tb.N**		**Tb.Sp (mm)**		**Tb.1/N.SD**		BV/TV**BV/TV**		**Ct.Po (%)**	
	*β*	*P*	*β*	*P*	*β*	*P*	*β*	*P*	*β*	*P*	*β*	*P*
Age, years	0.503	**0.047**	−0.006	0.057	0.007	**0.042**	0.003	0.164	0.000	0.473	0.041	**0.044**
Female	−3.866	0.507	−0.206	**0.007**	0.171	**0.028**	−0.030	0.674	−0.026	0.059	−0.902	0.055
BMI	1.818	**0.003**	0.007	0.353	−0.008	0.303	0.004	0.543	0.004	**0.002**	0.063	0.182
Grip strength	–	–	–	–	–	–	−0.011	**0.002**	–	–	–	–
Duration, years	−0.162	0.537	−0.005	0.154	0.005	0.134	0.002	0.496	−0.001	**0.037**	−0.010	0.647
DAS28	0.462	0.742	−0.006	0.766	0.009	0.660	−0.019	0.245	−0.001	0.721	−0.012	0.917
HAQ	–	–	−0.007	0.899	−0.001	0.981	0.030	0.498	–	–	–	–
HCQ	–	–	–	–	–	–	–	–	0.021	0.071	–	–
TWH	–	–	–	–	–	–	–	–	–	–	0.861	**0.014**

Multivariable linear regression analysis was performed to identify potential factors associated with HRpQCT parameters at the distal tibia. Results for different parameters are presented in separate columns. *β β*-coefficient, *TWH* tripterygium wilfordii Hook

Regarding volumetric BMD, trabecular vBMD at the distal radius was negatively related to advanced age (*p* = 0.010), female sex (*p* = 0.005), low BMI (*p* = 0.009), and disease duration (*p* = 0.010). Cortical vBMD was negatively associated with age (*p* < 0.001), disease duration (*p* = 0.001), and activity (*p* = 0.049). By contrast, at distal tibia, only demographic features were correlated with vBMD. Treatment with HCQ was associated with higher total vBMD at both bone sites.

As for bone microarchitecture, advanced age was related to impairment of almost all parameters. Female sex was associated with impaired trabecular microstructure, thinner cortex, and lower cortical porosity (*p* = 0.001). BMI was positively correlated with trabecular fraction, number, and thickness. RA duration and activity were related to compromised trabecular microstructure at the distal radius but not the distal tibia. Functional disability was correlated to thinner cortical bone (*p* = 0.037). Ever having been treated with glucocorticoids showed no significant correlation with any parameters, while ever having been treated with *Tripterygium wilfordii Hook* (a traditional Chinese herbal medicine used an immunosuppressive agent) was related to increased cortical porosity.

## Discussion

Multiple studies have demonstrated increased risk for osteoporosis and fractures among patients with RA [2, 3]. However, the vast majority of literature on this subject has been focused on aBMD of the axial skeleton measured with DXA. This methodology does not directly take into account alterations in bone microarchitecture, which contributes importantly to bone strength [9]. As a novel technology, HRpQCT depicts high-resolution images of cortical and trabecular bone with low radiation exposure, providing rich information regarding bone geometry, volumetric BMD, microarchitecture, and biomechanical properties [12]. It has been applied over the past decade to assess local bone erosions and generalized bone loss in RA [19, 30]. However, previous studies have focused on the hands and distal radius, which may be directly affected by inflammatory arthritis, confounding the evaluation of generalized osteoporosis. In the present study, we applied HRpQCT for the first time among patients with RA in mainland China, investigating BMD and microstructural alterations at both the distal radius and tibia.

Osteoporosis in RA has been attributed to chronic systemic inflammation, reduced physical activity, vitamin D deficiency, and use of glucocorticoids [2]. Overexpressed pro-inflammatory cytokines result in imbalanced bone remodeling, increased bone resorption, and further local and systemic osteoporosis [31]. Compared with healthy controls, patients with RA in our study showed widespread alterations in bone geometry, density, and microstructure at both the distal radius and tibia, which included increase in bone area and cortical perimeter, as well as decline in cortical vBMD and thickness. Enlarged bone area and perimeter has previously been hypothesized to be a compensatory mechanism for cortical thinning to restore bone strength [18]. A trend of generalized impairment in trabecular microarchitecture, characterized by decreased trabecular volume fraction, number, enlarged separation, and inhomogeneity, was also shown in our study, consistent with existing reports [18–21]. Compared with previous studies, our study included more older patients and post-menopausal women, which may have led to low-bone quality among both patients and controls, diminishing the differences between them. The relationship between RA and cortical porosity remains controversial. In a study by Zhu and colleagues, cortical porosity was the most dramatically changed microarchitectural parameter in RA [21]. However, it was not significantly altered in our study, nor in a study by Kocijan et al. [18].

To our knowledge, our study is the first to apply HRpQCT to evaluate the distal tibia in patients with RA. Prior studies have primarily focused on assessing the distal radius, which is adjacent to the wrist joint, a common site of inflammation in RA. These studies have demonstrated changes at the distal radius involving a wide range of densitometric and microstructural parameters, which were correlated with RA duration, disease activity, and wrist deformity [21, 32]. However, it has not been clear to what degree these changes reflect systemic changes versus localized periarticular change. Our findings showed that the distal tibia in patients with RA shared similar alterations with the radius in bone geometry, density, and microarchitecture, but was less affected by RA-related factors (e.g., disease duration and activity) than the radius. Given ankle joint inflammation was comparatively rare in our study population, our data suggest that the changes observed represent systemic bone alterations, and help explain the increased fracture risk in patients with RA.

Several demographic and clinical factors were associated with HRpQCT parameters in the RA group. Age, female sex, and low BMI were related to smaller cortical area, reduced BMD, and impairment of microarchitecture, consistent with previous studies [18, 21]. ACPA showed no correlation with any measures in our study, unlike the report by Stemmler et al. [19]. They compared 98 ACPA-positive patients with 84 ACPA-negative patients and found an adverse impact of ACPA on bone properties. This difference in results might be due to the small number of ACPA negative patients in our study. Disease duration and activity correlated with bone density and quality at the distal radius, while their impacts at the distal tibia were relatively scarce. Unlike healthy controls, compared with women, men with RA showed no advantages in bone parameters, which was similar to previous reports [19], indicating that impact of RA might be more pronounced among male patients. Reproductive factors were analyzed among women with RA, supporting the protective effect of estrogen on bone [33–35].

Although the association between impaired bone microarchitecture and fractures has been widely demonstrated in studies applying HRpQCT in the general population [16, 36], data from patients with RA are still limited. Kocijan et al. found that no individual microarchitectural parameters could discriminate between patients with and without fractures [18]. Stemmler and colleagues demonstrated a correlation between bone strength and fractures through micro-finite element analysis (μFEA), a technique based on HRpQCT that mathematically models stiffness and failure load to reveal bone biomechanical properties [37]. In this study, we focused on fractures occurring after RA diagnosis to reduce confounding by risk factors present prior to the onset of RA. In addition to decreased vBMD, patients with fractures showed generalized impairment in bone microarchitecture, and the trend of lower stiffness and failure load further suggested a decline in bone strength. HRpQCT has helped elucidate bone alterations in a variety of conditions; however, its application as a clinical assessment tool is still under study. Because HRpQCT devices are currently not widely available, the technique is mainly used for research purposes. Recently published studies have suggested potential value of HRpQCT parameters (e.g., failure load, vBMD, cortical area, trabecular number, and thickness) to improve prediction of fractures in general population [16, 38–40]. However, findings from the general population may not be directly translatable to populations with rheumatic diseases due to the impact of joint inflammation on adjacent distal limbs. Therefore, larger, dedicated studies among patients with rheumatic disease are necessary to help define the clinical applications of HRpQCT in this population.

With regard to medications, the association between GCs (duration and cumulative dose) and incident fractures in RA has been reported in numerous studies [41–43]. In our study, current GC users were shown to have decreased vBMD and impairment in trabecular and cortical microstructure, especially at the distal tibia, remote from commonly involved joints in RA. Our results showed no impact of dose of GCs, which may be due to the low average dose of GC in our study. Cumulative doses of GCs were not calculated, due to the concerns for recall bias. The association between HRpQCT parameters and other common therapies for RA remains to be elucidated. Biologic DMARDs have been reported to be protective against bone loss in RA due to their anti-inflammatory effects [42, 44], while their effects against microarchitectural impairment and incident fractures require further investigation [45]. Since almost all patients in our study were taking calcium and vitamin D, and few were treated with bisphosphonates, the effects of anti-osteoporotic treatment were not analyzed.

Our study has several limitations. First, the sample size was relatively small, which limits the statistical power of subgroup analyses by fracture status, sex, and other clinical parameters such as ACPA positivity, duration, and dose of GC. Therefore, our findings should be confirmed in larger studies. Second, the healthy control group was not recruited concurrently with the RA group. However, recruitment occurred in the same hospital only 2 years apart, images were obtained by the same technician on the same HRpQCT machine, and participants were matched to maximize comparability. Third, we did not perform μFEA to all patients. We hope to incorporate this in future research and provide additional information regarding bone strength and mechanical properties [46]. Fourth, since our study was cross-sectional in design, conclusions cannot be drawn with regard to causality from the associations observed between risk factors, bone parameters, and fractures.

## Conclusions

In summary, out study shows that patients with RA have reduced bone mineral density and impaired microarchitecture in both trabecular and cortical bone compared with healthy individuals. Alterations at the distal tibia and distal radius were similar, supporting the systemic influences of RA on the bone. Traditional risk factors for osteoporosis as well as RA-related factors are correlated with bone impairment. Patients with fragility fractures have more severely compromised bone parameters than those without. This cross-sectional study demonstrates the need for future larger prospective studies to better understand factors influencing microarchitectural deterioration in RA. Such data will be of vital importance for elucidating the contribution of these parameters to incident fractures and ultimately for the prevention of fractures in this vulnerable population.

## Supplementary Information

**Additional file 1: Supplemental Figure 1.** Flowchart of study participants. **Supplemental Table 1.** HRpQCT measures among patients with RA and healthy controls, by sex. **Supplemental table 2.** Comparison of HRpQCT parameters between women with RA, by menstrual status. **Supplemental Table 3.** Comparison of HRpQCT parameters between patients with RA, by GC therapy status.

## Data Availability

The datasets used and/or analyzed during the current study are available from the corresponding author on reasonable request.

## Abbreviations

ACPA: Anti-citrullinated protein antibodies
bDMARD: Biological DMARD
BMD: Bone mineral density
BV/TV: Trabecular bone volume fraction
CRP: C-reactive protein
CDAI: Clinical Disease Activity Index
csDMARD: Conventional synthetic disease-modifying antirheumatic drugs
Ct.Ar: Cortical bone area
Ct.Pm: Cortical perimeter
Ct.vBMD: Cortical vBMD
Ct.Th: Cortical thickness
Ct.Po: Intra-cortical porosity
DAS28: Disease activity score 28
ESR: Erythrocyte sedimentation rate
GC: Glucocorticoid
HAQ-DI: Health Assessment Questionnaire Disability Index
HCQ: Hydroxychloroquine
HRpQCT: High-resolution peripheral quantitative computed tomography
LEF: Leflunomide
MTX: Methotrexate
OP: Osteoporosis
RA: Rheumatoid arthritis
RF: Rheumatoid factor
SDAI: Simplified Disease Activity Index
Tt.Ar: Total bone area
Tb.Ar: Trabecular bone area
Tt.vBMD: Total volumetric BMD
Tb.vBMD: Trabecular vBMD
Tb.Th: Trabecular thickness
Tb.N: Trabecular number
Tb.Sp: Trabecular separation
Tb.1/N.SD: Inhomogeneity of network
μFEA: Micro-finite element analyses
