# Supplementary material for: Bone mineral density and microarchitecture among Chinese patients with rheumatoid arthritis: a cross-sectional study with HRpQCT
Source: Arthritis Res Ther. 2021 Apr 24;23:127. doi: 10.1186/s13075-021-02503-0 (PMC8067377; doi:10.1186/s13075-021-02503-0)
Supplement: Supplementary file 1 — Additional file 1: Supplemental Figure 1. Flowchart of study participants. Supplemental Table 1. HRpQCT measures among patients with RA and healthy controls, by sex. Supplemental table 2. Comparison of HRpQCT parameters between women with RA, by menstrual status. Supplemental Table 3. Comparison of HRpQCT parameters between patients with RA, by GC therapy status. [file 13075_2021_2503_MOESM1_ESM.docx]

**Supplemental Figure 1. Flowchart of study participants**

OPF = osteoporotic fracture; RA = rheumatoid arthritis; PUMCH = Peking Union Medical College Hospital

**Supplemental Table 1. HRpQCT measures among patients with RA and healthy controls, by sex**

|  | Control Group | | RA Group | |
| --- | --- | --- | --- | --- |
|  | Male (n= 12) | Female (n= 69) | Male (n=12 ) | Female (n= 69) |
| Radius |  |  |  |  |
| Tt.Ar,mm^2^ | 383.7±48.7*† | 262.9±51.9*† | 339.2±48.5*† | 238.1±34.7*† |
| Ct.Ar,mm^2^ | 74.6±15.4* | 54.6±11.3* | 71.9±7.0* | 53.5±9.3* |
| Tb.Ar,mm^2^ | 313.7±46.8*† | 212.2±54.8*† | 271.6±51.0*† | 188.0±36.0*† |
| Ct.Pm,mm^2^ | 84.8±6.9* | 68.6±8.1*† | 79.6±6.6* | 64.0±4.7*† |
| Tt.vBMD | 254.3±55.4† | 259.2±87.4 | 300.6±44.5† | 281.5±73.6 |
| Ct.vBMD | 839.2±85.9 | 890.5±114.3 | 887.7±51.7 | 915.9±59.2 |
| Tb.vBMD | 116.0±30.5† | 90.7±43.6 | 143.3±32.4*† | 100.3±40.8* |
| BV/TV | 0.172±0.042 | 0.139±0.056 | 0.210±0.048* | 0.152±0.054* |
| Tb.Th,mm | 0.232±0.009* | 0.217±0.020* | 0.232±0.014* | 0.216±0.016* |
| Tb.N,1/mm | 1.254±0.273 | 1.058±0.327 | 1.357±0.248* | 1.111±0.288* |
| Tb.Sp,mm | 0.783(0.675-1.009) | 0.902(0.748-1.154) | 0.694(0.605-0.891)* | 0.850(0.733-1.158)* |
| Tb.1/N.SD | 0.327(0.277-0.466) | 0.380(0.288-0.559) | 0.290(0.227-0.383) | 0.324(0.273-0.549) |
| Ct.Th,mm | 1.072±0.239 | 0.962±0.257 | 1.105±0.143* | 0.989±0.190* |
| Ct.Po,% | 1.30(0.78-1.85) * | 0.50(0.30-0.90) * | 1.25(0.60-1.73) * | 0.50(0.30-0.70) * |
| Tibia |  |  |  |  |
| Tt.Ar,mm^2^ | 904.9±79.7* | 678.5±103.1* | 833.1±105.2* | 652.5±87.4* |
| Ct.Ar,mm^2^ | 134.7±29.5* | 96.1±23.7*† | 129.9±23.8* | 103.5±19.1*† |
| Tb.Ar,mm^2^ | 776.4±84.6* | 587.3±107.7* | 709.2±110.2* | 554.2±93.0* |
| Ct.Pm,mm^2^ | 118.3±5.4* | 101.3±7.6* | 113.1±7.3* | 99.2±6.9* |
| Tt.vBMD | 228.8±43.0 | 216.2±66.5 | 255.2±48.9 | 233.8±52.0 |
| Ct.vBMD | 820.3±85.4 | 866.8±89.4 | 845.8±77.7 | 873.6±77.2 |
| Tb.vBMD | 125.8±27.1 | 106.3±37.6 | 144.7±36.8* | 112.2±34.5* |
| BV/TV | 0.200±0.034 | 0.175±0.048 | 0.226±0.048* | 0.182±0.044* |
| Tb.Th,mm | 0.248±0.012 | 0.240±0.020 | 0.248±0.017 | 0.238±0.019 |
| Tb.N,1/mm | 1.264±0.277* | 1.090±0.218* | 1.258±0.283 | 1.113±0.186 |
| Tb.Sp,mm | 0.814(0.710-0.864) | 0.885(0.808-1.075) | 0.801(0.708-0.978) | 0.882(0.801-1.002) |
| Tb.1/N.SD | 0.339(0.285-0.371) | 0.381(0.306-0.495) | 0.338(0.284-0.398) | 0.349(0.323-0.415) |
| Ct.Th,mm | 1.365±0.277* | 1.130±0.300*† | 1.373±0.296 | 1.235±0.255† |
| Ct.Po,% | 4.30(2.60-5.00) * | 2.20(1.20-3.45) * | 3.85(2.20-5.38) * | 2.70(1.40-3.80) * |

*p＜0.05 in the comparison between men and women for participants within the same group

† p＜0.05 in the comparison between the RA group and Control group for participants of the same sex

**Supplemental table 2. Comparison between women with RA, by menstrual status**

|  | Women with RA  N=69 | Premenopausal  n=18 | Postmenopausal  n=51 | P |
| --- | --- | --- | --- | --- |
| Age | 57.0±8.8 | 44.6±3.8 | 61.0±5.5 | **＜0.001** |
| Radius | | | | |
| Tt.Ar,mm^2^ | 262.9±51.9 | 241.6±39.7 | 269.9±53.8 | 0.050 |
| Ct.Ar,mm^2^ | 54.6±11.3 | 62.7±5.9 | 51.9±11.3 | **＜0.001** |
| Tb.Ar,mm^2^ | 212.2±54.8 | 182.3±39.7 | 221.9±55.8 | **0.009** |
| Ct.Pm,mm^2^ | 68.6±8.1 | 64.1±5.2 | 70.1±8.4 | **0.007** |
| Tt.vBMD | 259.2±87.4 | 346.7±68.6 | 230.6±72.9 | **＜0.001** |
| Ct.vBMD | 890.5±114.3 | 992.4±39.9 | 857.2±111.1 | **＜0.001** |
| Tb.vBMD | 90.7±43.6 | 121.5±43.1 | 80.7±39.2 | **0.001** |
| BV/TV | 0.139±0.056 | 0.176±0.063 | 0.128±0.049 | **0.002** |
| Tb.Th,mm | 0.217±0.020 | 0.215±0.017 | 0.217±0.021 | 0.759 |
| Tb.N,1/mm | 1.058±0.327 | 1.277±0.278 | 0.986±0.312 | **0.001** |
| Tb.Sp,mm | 0.902(0.748-1.154) | 0.695(0.651-0.999) | 0.991(0.832-1.374) | **0.001** |
| Tb.1/N.SD | 0.380(0.288-0.559) | 0.263(0.233-0.410) | 0.421(0.316-0.670) | **0.003** |
| Ct.Th,mm | 0.962±0.257 | 1.150±0.117 | 0.900±0.261 | **＜0.001** |
| Ct.Po,% | 0.50(0.30-0.90) | 0.20(0.20-0.35) | 0.60(0.40-0.90) | **＜0.001** |
| Tibia | | | | |
| Tt.Ar,mm^2^ | 678.5±103.1 | 633.7±95.8 | 693.4±101.9 | **0.038** |
| Ct.Ar,mm^2^ | 96.1±23.7 | 112.4±21.4 | 90.7±22.0 | **0.001** |
| Tb.Ar,mm^2^ | 587.3±107.7 | 524.9±97.8 | 608.1±103.6 | **0.005** |
| Ct.Pm,mm^2^ | 101.3±7.6 | 98.1±7.5 | 102.4±7.4 | **0.040** |
| Tt.vBMD | 216.2±66.5 | 275.3±68.3 | 196.5±53.4 | **＜0.001** |
| Ct.vBMD | 866.8±89.4 | 961.7±66.9 | 835.1±72.1 | **＜0.001** |
| Tb.vBMD | 106.3±37.6 | 123.5±43.4 | 100.6±34.1 | **0.029** |
| BV/TV | 0.175±0.048 | 0.193±0.058 | 0.170±0.044 | 0.078 |
| Tb.Th,mm | 0.240±0.020 | 0.232±0.021 | 0.243±0.019 | **0.049** |
| Tb.N,1/mm | 1.090±0.218 | 1.237±0.190 | 1.041±0.206 | **0.001** |
| Tb.Sp,mm | 0.885(0.808-1.075) | 0.761（0.679-0.934） | 1.008（0.823-1.119） | **0.002** |
| Tb.1/N.SD | 0.381(0.306-0.495) | 0.286（0.274-0.392） | 0.409（0.331-0.533） | **0.001** |
| Ct.Th,mm | 1.130±0.300 | 1.341±0.292 | 1.060±0.271 | **0.001** |
| Ct.Po,% | 2.20(1.20-3.45) | 1.00（0.75-2.00） | 2.50（1.60-3.80） | **＜0.001** |

**Supplemental Table 3. Comparison between patients with RA, by GC therapy status**

|  | Radius | | | Tibia | | |
| --- | --- | --- | --- | --- | --- | --- |
|  | Currently receiving GCs  n=37 | Not currently receiving GCs  n=44 | P | Currently receiving GCs  n=36 | Not currently receiving GCs  n=44 | P |
| Tt.vBMD | 238.7 ± 76.3 | 275.1 ± 85.8 | **0.049** | 203.6 ± 60.9 | 230.0 ± 63.8 | 0.064 |
| Ct.vBMD | 850.2 ± 119.1 | 910.4± 98.0 | **0.015** | 831.7 ± 79.9 | 882.8± 91.8 | **0.011** |
| Tb.vBMD | 89.7 ± 38.9 | 98.5 ± 45.8 | 0.355 | 105.2 ± 34.4 | 112.6 ± 38.7 | 0.370 |
| BV/TV | 0.140 ± 0.048 | 0.148 ± 0.061 | 0.573 | 0.177 ± 0.042 | 0.181 ± 0.051 | 0.712 |
| Tb.Th, mm | 0.222 ± 0.014 | 0.216 ± 0.022 | 0.186 | 0.248 ± 0.018 | 0.236 ± 0.018 | **0.005** |
| Tb.N, 1/mm | 1.033 ± 0.330 | 1.132 ± 0.320 | 0.179 | 1.055 ± 0.265 | 1.166 ± 0.195 | **0.034** |
| Tb.Sp, mm | 0.915（0.797-1.453） | 0.875（0.689-1.081） | 0.152 | 1.008（0.824-1.121） | 0.833（0.725-1.009） | **0.016** |
| Tb.1/N.SD | 0.426（0.317-0.722） | 0.358（0.249-0.476） | 0.084 | 0.417（0.339-0.544） | 0.337（0.284-0.441） | **0.003** |
| Ct.Th, mm | 0.947 ± 0.282 | 1.004 ± 0.232 | 0.325 | 1.114 ± 0.321 | 1.207 ± 0.293 | 0.179 |
| Ct.Po,% | 0. 80(0.40-1.20) | 0. 50(0.30-0.90) | **0.038** | 2.65(1.90-4.40) | 2.05(1.05-3.30) | **0.048** |
